# Expression of the Costimulatory Molecule B7-H4 in the Decidua and Placental Tissues in Patients with Placental Abruption

**DOI:** 10.3390/biomedicines10040918

**Published:** 2022-04-16

**Authors:** Monika Bączkowska, Magdalena Maria Dutsch-Wicherek, Ewa Przytuła, Jan Faryna, Cezary Wojtyła, Mohamed Ali, Anna Knafel, Michał Ciebiera

**Affiliations:** 1 Centre of Postgraduate Medical Education, Second Department of Obstetrics and Gynecology, 01-809 Warsaw, Poland; monika.baczkowski@gmail.com (M.B.); annaknafel@gmail.com (A.K.); 2 Centre of Postgraduate Medical Education, Department of Psychiatry, 01-809 Warsaw, Poland; dutsch.wicherek@gmail.com; 3 Department of Pathology, Bielański Hospital, 01-809 Warsaw, Poland; ewa.przytula.szpital@gmail.com (E.P.); janfaryna@poczta.fm (J.F.); 4 International Prevention Research Institute-Collaborating Centre, Calisia University, 62-800 Kalisz, Poland; cezary.wojtyla@gmail.com; 5 Clinical Pharmacy Department, Faculty of Pharmacy, Ain Shams University, Cairo 11566, Egypt; mohamed.aboouf@pharma.asu.edu.eg

**Keywords:** B7-H4, pregnancy, decidua, placental abruption, immunology, cytotoxicity, maternal–fetal interface

## Abstract

B7 homolog 4 protein (B7-H4), a member of the B7 family, is a immunomodulatory membrane protein. The aim of the study was to evaluate the expression of this protein in the decidua and placental tissues in case of placental abruption (PA) compared to cases of retained placental tissue (RPT) and controls. Tissue samples were obtained from 47 patients with PA, 60 patients with RPT, and 41 healthy controls. The samples were stained for B7-H4 expression, analyzed by an expert pathologist, and a semi-quantitative scale was applied. A statistical analysis revealed that the expression of B7-H4 was significantly higher in the decidua in PA samples compared to samples from patients with RPT (*p*-value < 0.001) and healthy controls (*p*-value < 0.001). The expression of B7-H4 in the placental chorionic villus was significantly higher in PA samples in relation to samples from healthy controls (*p*-value < 0.001) but not in relation to RPT samples (*p*-value = 0.0853). This finding suggests that B7-H4 might play an important role in mechanisms restoring reproductive tract homeostasis. Further research is necessary in regard to the role of B7-H4 in PA.

## 1. Introduction

The state of pregnancy is a unique balance between the activation and inhibition of the immune system in the female reproductive tract, which supports the existence and development of the fetus inside the mother’s body [1]. Remarkably, during pregnancy, the immune system is challenged to permit fetal growth in the uterus, while continuing to eliminate attacking pathogens [2].

One of the most important regulators of this balance is the decidua. The human decidua is a highly specialized tissue with many unique regulatory properties. The decidua provides complex immunological protection as well as wide nutritional support to the newly developing life [3]. The decidual cells coexist with a full range of immune cells that consist of a complex, branched system with diverse connections [1,4,5,6]. They include decidual natural killer cells (dNK) [1,7], natural killer T (NKT) cells [8], regulatory T cells (Tregs) [9,10,11], monocytes, macrophages [4,7], lymphocytes, and dendritic cells (DCs) [12]. They also influence the infiltration and activity of one another as well as other cells [13,14]. The coexistence of the decidual and immune cells is feasible due to the development of resistance to the immune-mediated apoptosis of the endometrial cells [3,15,16].

Placental abruption (PA) is defined as a complete or partial separation of the placenta from the uterine wall during pregnancy, with the fetus still being present in the uterine cavity. It is a serious perinatal complication and one of the leading causes of second- and third-trimester bleeding [17,18,19]. Placental abruption occurs in about 1% of cases [20], with the mortality rate of about 10% [21]. Numerous pathophysiological notions are linked to PA, including reduced uteroplacental blood flow [22,23], decidual vasculopathy [24], endothelial cell dysfunction, lack of adequate trophoblastic cell invasion and impaired angiogenesis [25,26,27], thrombosis [28], bacterial infection [29,30,31], chronic inflammation [32,33,34,35,36], hemorrhage [12,35,37,38,39,40,41,42], or genetic predisposition [26,43,44,45]. Notably, the disruption of the uterine cavity may increase the risk of PA [46]. One of the most interesting contemporary perspectives concerning the pathophysiology of PA is the thought of PA as an immunological process taking place locally in the decidua. The significance of the decidual immunomodulatory activity was confirmed in several studies [12,47,48]. Membrane proteins expressed by the decidual cells modulate the maternal immune system activity and participate in the commencement of labor. Available data suggest that PA is a result of the accumulation of cytotoxic immune cells (i.e., neutrophils, macrophages) [35] accompanied by the insufficiency of decidual suppressive activity [12].

B7 homolog 4 protein–B7-H4 (AKA B7S1 or V-set domain containing T-cell activation inhibitor–VCTN1) is a costimulatory transmembrane molecule and a member of the regulatory membrane molecules B7 family [49,50,51,52,53]. The B7 family plays an essential role in maintaining tolerance to the fetus [50]. It is one of the most characterized and widely distributed signaling molecule superfamily, exerting both stimulatory and inhibitory effects via stimulatory and inhibitory receptors, respectively, on T cells [54,55]. B7-H4 was initially described in 2003 [56,57,58], has only an inhibitory receptor and, thus, is responsible for the negative regulation of T-cell-mediated immune responses [49,50,52,53,59,60,61,62]. Notably, B7-H4 only binds to activated T-cells and subsequently inhibits T-cell proliferation by cell cycle arrest apart from inhibition of the production of proinflammatory cytokines [56,57,58,63,64,65]. Conversely, it inhibits neutrophil infiltration and suppresses Th1 immune response [50]. 

B7-H4 presents with two functional isoforms—the soluble form (sB7-H4) and the membrane-bound form. The source and functional mechanisms of the soluble form of B7-H4 remains obscure [51,53,66,67,68,69,70,71,72,73]. However, there is growing evidence that sB7-H4 acts as a T-cell-negative regulatory molecule, similar to cell-associated B7-H4 [51,52,74,75,76,77]. The inhibitory B7-H4 receptor is undetermined [51], but some recent studies have shown that it binds to the soluble semaphorin (Sema) family member Sema3a protein [78]. The expression of B7-H4 is strictly controlled in peripheral tissues at the transcriptional level, B7-H4 mRNA being widely expressed, while the presence of the B7-H4 protein is mostly limited to the reproductive tract tissues and selected cancers [49,56,57,58,61,62,67,79]. It is mainly present on the antigen presenting cell (APC)–macrophages and DCs [51,80]. The expression of B7-H4 on the APCs varies in different pregnancy pathologies [52,53,81,82]. 

Importantly, B7-H4 is involved in immunological changes associated with the spontaneous onset of labor as well as with several adverse perinatal outcomes. Its expression is higher during labor than during pregnancy, but it does not change during the course of labor [80]. There is growing evidence that B7-H4 is responsible for the modulation of the immune response during labor and restoring the homeostasis of the reproductive tract after labor [51]. Higher sB7-H4 or B7-H4 expression was described in cases of preterm premature rupture of membranes (PPROM) [52]–the rupture of the amniotic sac before the onset of labor, occurring before 37 weeks of pregnancy [83]. It was also described in case of chorioamnionitis [52], hemolysis, elevated liver enzymes and low platelets (HELLP) syndrome [82], or preeclampsia (PE) [53,81,82,84]. PE is a disorder of pregnancy characterized by high blood pressure and concomitant signs of damage to other organs, with the liver and kidneys being the most commonly affected [85]. All these pregnancy complications are associated with exaggerated immune system activity, with the pathological Th1 cell predominance [86]. According to available data, the disruption of the immunological processes starts long before the visible outcomes (e.g., PE, PPROM or PA), as the increased amount of sB7-H4 was found in such cases as far back as in the first trimester of pregnancy [52,53]. It supports the concept of the induction failure of appropriate maternal immune tolerance and an exaggerated systemic maternal inflammatory response occurring in such cases [87,88,89,90,91]. The costimulatory molecule B7-H4 seems to answer this inflammatory process on the maternal–fetal interface from the very beginning, much before its visible manifestations.

The aim of this study was to explore B7-H4 expression in patients with PA and its significance to the process of placental detachment.

## 2. Materials and Methods

The study was performed retrospectively at the II Department of Obstetrics and Gynecology, Centre of Postgraduate Medical Education—a tertiary perinatal care center based in Warsaw, Poland. The local Ethics Committee approved the study (approval number 129/PB/2020).

### 2.1. Patients

We included cases from January 2017 to December 2019, for which the tissue material was available in the archives of the pathology department. We divided the identified cases into three groups: samples from patients diagnosed with PA, samples from patients diagnosed with retained placental tissue (RPT), and samples collected for other reasons (fetal growth restriction, threatening fetal asphyxia, breech presentation, lack of progress in labor, previous cesarean section or without a specified cause). The necessary clinical characteristics of the patients were extracted from the available hospital database. Patients with incomplete medical history were excluded from the study.

We included a total of 148 patients; 47 of them were patients with PA, 60 of them were patients with RPT, and 41 were healthy controls. PA diagnosis was based on the clinical symptoms including rapidly developing uterine tenderness, abdominal pain, severe vaginal bleeding/hemorrhage, and/or fetal distress. In all cases, the diagnosis of PA was first confirmed by the presence of a retroplacental clot and then was retrospectively confirmed in the tissue examined by a pathologist. All cases of diagnosed placental abruption were taken into consideration, despite the fact of the presence of obvious external bleeding [40], the percentage of separated placenta [92], the site of abruption [93], or the grade of the placental abruption [94,95]. The RPT diagnosis was based on the incomplete placental tissue after placental delivery and the presence of RPT inside the uterine cavity following the third stage of labor. It was also retrospectively confirmed by a pathologist in the examined tissues. We excluded patients diagnosed with atony or subatony, as in such cases bleeding could also be caused by a reason different than RPT. 

All the participants were of Polish ethnicity. The characteristics of the studied groups are presented in Table 1.

### 2.2. Tissue Samples and Immunohistochemistry

The selected tissue samples were retrieved from the Department of Pathology, Bielański Hospital, Warsaw, Poland. The paraffin-embedded placental chorionic villous and decidual tissue samples were evaluated by an expert pathologist who subsequently selected material sufficient for further analysis. The chosen samples were cut using the microtome into 3 μm slices and sent for immunohistochemical processing.

Immunohistochemical analysis was performed manually in the Department of Pathology, Bielański Hospital, with the Ultravision LPValue Detection System (Thermo Scientific Lab Vision Corporation, Fremont, CA, USA). The visualization of reaction products was performed with 3,3′-diaminobenzidine (DAB+) chromogen (DAKO, Carpinteria, CA, USA) used for 10 min at room temperature, receiving a gold–brown color of the final product. In the next step, the sections were counterstained with Meyer’s hematoxylin and mounted in glycergel. According to the recommendations of the producer, the specificity of the B7-H4 antibody was tested with a specimen of ductal breast cancer, constituting a positive control for B7-H4. The results obtained from the control were in accordance with the producer’s specification.

Afterwards, the slides were washed in tris-buffered saline (TBS) plus 0.025% Triton X-100 and blocked for 2 h at room temperature in 10% normal serum with 1% bovine serum albumin (BSA) in TBS. The slides were drained and incubated with the primary antibody, rabbit polyclonal B7-H4 (ABCAM; Cambridge Biomedical Campus, Cambridge, UK, Catalog No. EPR20236), in 1:100 dilution in TBS with 1% BSA. The incubation with the primary monoclonal antibody took place in a humidified chamber overnight at 4° Celsius. Then, the slides were rinsed twice for 5 min with TBS plus 0.025% Triton and submitted to 0.3% H_2_O_2_ in TBS for 15 min. Subsequently, the application of the secondary enzyme-conjugated antibody diluted in TBS with 1% BSA took place. The incubation lasted for 1 h at room temperature. The slides were developed with chromogen for 10 min at room temperature and then rinsed in running tap water and, subsequently, counterstained with hematoxylin.

The decidua is the endometrial tissue of the uterine cavity remodeled during the pregnancy under the influence of hormones. The tissue consists of monomorphic stromal cells closely adjacent to each other, expressing certain features of the epithelial tissue. All of the examined placental samples were collected from patients in the third trimester of pregnancy. The villi of the placenta in the last trimester of pregnancy are composed of connective stromal tissue surrounded by a layer of syncytiotrophoblast with thin-walled blood vessels located on the circumference of the villi under the syncytiotrophoblast. Sinus-type vessels are present in the stroma near the syncytiotrophoblast. All the obtained samples were evaluated by an expert pathologist and qualified as typical and appropriate for further analysis.

**Figure 1:**
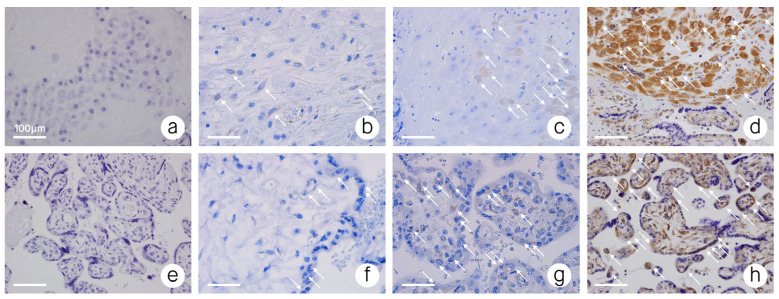
B7-H4 immunoreactivity; (**a**) decidua–stage 0–no B7-H4 immunoreactivity; (**b**) decidua–stage 1–low B7-H4 immunoreactivity; (**c**) decidua–stage 2–moderate B7-H4 immunoreactivity; (**d**) decidua–stage 3–high B7-H4 immunoreactivity; (**e**) placental chorionic villus–stage 0–no B7-H4 immunoreactivity; (**f**) placental chorionic villus–stage 1–low B7-H4 immunoreactivity; (**g**) placental chorionic villus–stage 2–moderate B7-H4 immunoreactivity; (**h**) placental chorionic villus–stage 3–high B7-H4 immunoreactivity. Objective magnification ×40. ↖—Points some of the cells expressing B7-H4.

**Figure 2:**
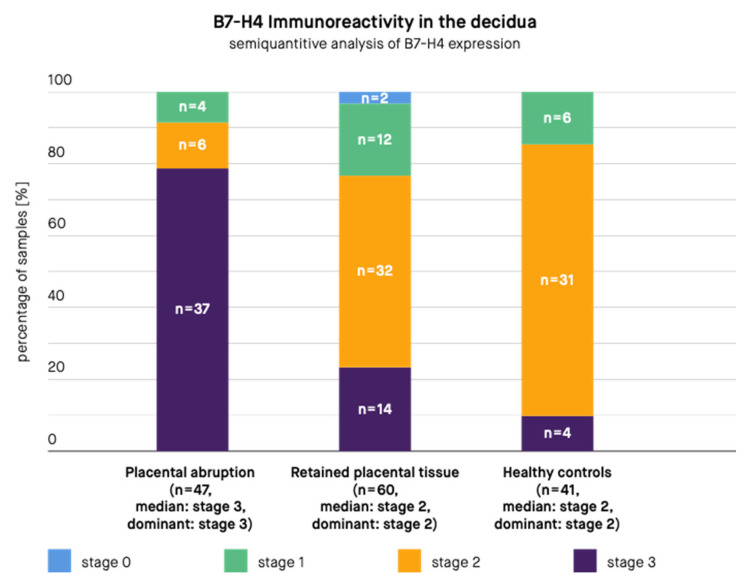
B7-H4 immunoreactivity in the decidua in samples from patients with placental abruption, retained placental tissue, and healthy controls. *n—Number of samples*.

**Figure 3:**
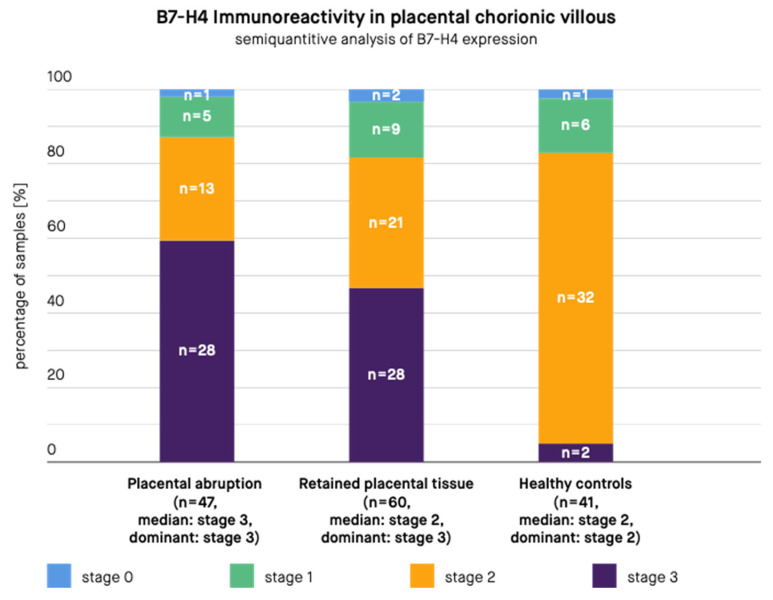
B7-H4 immunoreactivity in the placental chorionic villus in samples from patients with placental abruption, retained placental tissue, and healthy controls. *n—Number of samples*.

A semi-quantitative scale based on the previously published studies [12,82,96] was then applied to the obtained samples (Table 2, Figure 1). The usage of a semi-quantitative scale was necessary because of a significant difference in the staining patterns of cells. Therefore, calculating only the percentage of stained cells would lead to the loss of a significant part of information. The analysis was carried out by an expert pathologist. The number and staining pattern of B7-H4-positive placental chorionic villous and decidual basalis cells per one HPF (high power field–objective magnification ×40, Nikon Eclipse 50i Microscope; Nikon Corporation, Tokyo, Japan) was estimated. The calculation was made through the entire slides (at least 10 HPFs per sample). On the basis of this, the average percentage and staining pattern of B7-H4 positive cells was estimated and the semi-quantitative scale was applied. The criteria of the semi-quantitative scale are presented in Table 2. In case of the lack of reactivity in the whole sample or some reactivity present in <1% of cells the sample was qualified as “Stage 0”. In case of any staining pattern (low or high) present in only 1–20% of cells the sample was qualified as “Stage 1”. In case of a low staining pattern present in 21–50% of cells the sample was qualified as “Stage 2”. In case of a low staining pattern present in >50% of cells or high staining pattern present in >20% of cells the sample was qualified as “Stage 3”. Subsequently, the authors compared B7-H4 expression in PA samples with that in RPT samples and healthy control samples (Figure 2 and Figure 3).

### 2.3. Statistical Analysis

The normality of variable and group characteristics distributions were tested with the Shapiro–Wilk test. Regarding the group characteristics data, only the age was normally distributed. The ANOVA test and Kruskal–Wallis test were applied to analyze the differences between group characteristics. B7-H4 expression levels were found to be non-normally distributed. Statistical differences between groups were estimated using standard non-parametric tests (the Dunn’s test with Benjamini–Hochberg adjustment). Significance was accepted at *p* < 0.05. The R version 4.1.0 software was used for statistical analysis.

## 3. Results

A total of 148 women were enrolled in this study. The groups did not differ in terms of age, parity, and BMI, but inevitably differed in terms of gestational age and newborn weight and length. The control group had the highest rate of live births (Table 1). The presence of B7-H4 was confirmed in all decidual tissue samples from patients with PA (100%) and healthy controls (100%) and most of the samples from patients with RPT (97%). The presence of B7-H4 in placental chorionic villus was also confirmed in the majority of samples from patients with PA (98%), RPT (97%), and from healthy controls (98%). 

The results are presented in Figure 2 and Figure 3. According to the immunohistochemical images, B7-H4 expression was present in the stroma and syncytiotrophoblast of the placental chorionic villus, both in the cytoplasm and at the cell membrane. B7-H4 expression was also noted both in the cytoplasm and at the cell membrane of the decidua. The expression of the B7-H4 molecule in the decidua was significantly higher in case of PA compared to RPT (median in PA group: stage 3, dominant in PA group: stage 3, median in RPT group: stage 2, dominant in RPT group: stage 2, *p*-value < 0.001) and in case of PA compared to healthy controls (median in PA group: stage 3, dominant in PA group: stage 3, median in healthy controls group: stage 2, dominant in healthy controls group: stage 2, *p*-value < 0.001). The difference between the control group and RPT group was not significant (median in RPT group: stage 2, dominant in RPT group: stage 2, median in healthy controls group: stage 2, dominant in healthy controls group: stage 2, *p*-value = 0.3055). The expression of the B7-H4 molecule in the placental chorionic villus did not significantly differ in case of PA compared to RPT (median in PA group: stage 3, dominant in PA group: stage 3, median in RPT group: stage 2, dominant in RPT group: stage 3, *p*-value = 0.0853), but the difference remained significant in case of PA compared to healthy controls (median in PA group: stage 3, dominant in PA group: stage 3, median in healthy controls group: stage 2, dominant in healthy controls group: stage 2, *p*-value < 0.001) and in case of RPT compared to the control group (median in RPT group: stage 2, dominant in RPT group: stage 3, median in healthy controls group: stage 2, dominant in healthy controls group: stage 2, *p*-value = 0.0012).

## 4. Discussion

According to available data, labor might be considered to be the result of the cessation of maternal immune tolerance toward the fetus. The augmented decidual cytotoxic activity is one of the most important components of the processing cascade leading to the expulsion of the fetus [97,98]. 

At the molecular level, the process of the development of PA takes place locally on the maternal–fetal interface and is not reflected in the peripheral blood [99,100,101,102,103,104]. When the immunological processes in the decidual microenvironment take place in a proper order, placental detachment occurs after cervical ripening and uterine contractions resulting in the expulsion of the fetus [105]. The disruption of the molecular processes on the maternal–fetal interface may lead to the improper order of these events and PA [1,12,106,107].

Not only during labor, PA is also accompanied by an increase in the cytotoxic activity of lymphocytes [12,108,109,110]. It results from the alteration of the maternal immune tolerance toward fetal antigens [12,48,110,111,112] and the breakthrough of the pregnancy-specific Th_2_ domination [108,113]. During PA, the levels of immunomodulating membrane proteins on the maternal–fetal interface are reduced and immune cell infiltration is more intense than that during physiological labor [1,12,35,114].

**Figure 4:**
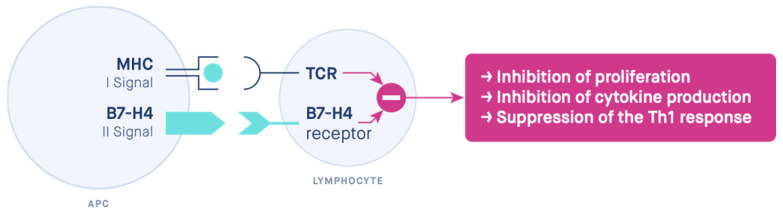
The two-signal hypothesis. The mechanism of action of the costimulatory molecule B7-H4. *MHC—major histocompatibility complex, TCR—T-cell receptor, APC—antigen-presenting cell*.

B7-H4 immunosuppressive functions seem to be similar to Tregs. Tregs also stimulate B7-H4 expression on macrophages [57], e.g., via the stimulation of APC production of interleukin 10 (IL-10) [115] and via macrophage sensitization to the action of interleukin 6 (IL-6) [116]. Moreover, IL-6 and IL-10 stimulate the expression of B7-H4 on macrophages [62,116,117]. It is possible that the fetus actively participates in this process by producing IL-6 [80,111]. B7-H4 inhibits interleukin 2 (IL-2) production through interfering with the activation of extracellular signal-regulated kinase (ERK), c-Jun N-terminal kinase (JNK), and protein kinase B (PKB, Akt) [118]. Being costimulatory molecules, the members of the B7 family determine the ultimate immune response by the transduction of the second signal [119] (Figure 4). As the antigen is recognized by the T-cell and merges the T-cell receptor (TCR) to the peptide-major histocompatibility complex (MHC), a co-signaling molecule is needed to transduce the second signal, thus controlling the effect of the stimulation and leading to activation or anergy [120,121,122].

To the best of our knowledge, this is the first study that analyzed B7-H4 immunoreactivity levels in the placental chorionic villus and decidual tissue samples from patients who were diagnosed with PA. Interestingly, B7-H4 expression in the decidual tissues turned out to be significantly higher in PA samples in relation to RPT samples and in healthy controls. The difference was also significant in the placental chorionic villus when comparing PA samples to healthy controls. This effect seemed to be contradictory to formerly investigated molecules responsible for the inhibition of maternal immune response, such as the receptor-binding cancer antigen expressed on SiSo cells (RCAS1) [1] or Tregs [80]. It compelled us to consider the phenomenon from a different perspective. As the augmentation of the immune response must be controlled and finally stopped, mechanisms functioning in the opposite direction have to start simultaneously. The activation of the maternal immune system is accompanied by opposite reactions leading to the restriction of the same activation. It enables the reproductive tract to return to the initial balance. In advanced labor, the total number and activity of immune cells in the decidua decreases [123], which supports the hypothesis of the self-limiting character of the process. B7-H4, as a T-cell activation inhibitor, seemed to be a part of this negative feedback loop. 

An important limitation of the presented study is its retrospective character and the unavoidable fact that we could use only the available data and tissue material. Ideally, we would like to obtain samples from healthy controls without any comorbidities, nor pregnancy or labor complications, to avoid the bias of this issues. However, tissue samples are not routinely collected in such cases. It would also be beneficial to have a control group at a more precisely matching pregnancy age, as the difference may exert some influence on the immune response in the decidua. Nonetheless, according to many previous data, the changes of the immune response in the decidua seem mainly related to pregnancy complications or the state of labor. Thus, we assumed the level of bias acceptable [1,12,97,98,99,100,101,102,103,104,105,108,110]. Moreover, regarding the availability of detailed patient data in hospital databases, retrospective studies frequently reveal significant scarcity. Nonetheless, the obtained results are encouraging in terms of continuing the studies in a prospective manner, to enable more detailed and reliable analyses.

The analyzed samples confirmed the involvement of the immunomodulatory molecule B7-H4 in the processes occurring on the maternal–fetal interface. B7-H4 seems to justify the prematurely increased decidual cytotoxic activity present in PA and is responsible for restoring reproductive tract balance. A focused look at the molecular basis of this clinically important pregnancy complication validates, with greater reliability, the significance of this investigated molecule in the placental detachment process. Considering the fact that PA results in perinatal deaths and numerous serious complications for both the mother and the child, it is critical to put effort to thoroughly understand the underlying mechanisms. Any progress that leads to the reduction of mortality and morbidity through the improvement of PA management is valuable.

## 5. Conclusions

Our study revealed the abnormal expression of B7-H4 in cases of PA in women of Polish ethnicity. According to this finding and previous data, a significant role of this molecule might be indicated in maintaining maternal immune system balance, which is disrupted in case of PA. Our results also indirectly confirm increased cytotoxic activity on the maternal–fetal interface in case of PA, which starts at the early beginning, far before the actual detachment of the placenta. More clinical and molecular studies are still necessary in this matter. The molecular mechanisms underlying PA pathogenesis are an interesting target for further research to better understand and, thus, develop the appropriate management of this serious perinatal complication.

## Figures and Tables

**Table 1:** Group characteristics.

Patients (*n* = 148)	Maternal Age ± SD (Year)	BMI ± SD (BMI)	Gestational Age ± SD (Week)	Parity Nulliparous (%)	Newborn Weight ± SD (g)	Newborn Length ± SD (cm)	Live Births (%)
Placental abruption (*n* = 47)	33 ± 5	26 ± 4	30 ± 4	44	1489 ± 865	40 ± 7	87
Retained placental tissue (*n* = 60)	31 ± 5	26 ± 4	32 ± 6	40	1959 ± 1128	44 ± 9	67
Healthy controls (*n* = 41)	32 ± 6	27 ± 6	37 ± 3	37	2851 ± 738	51 ± 4	95
*p*-value	0.368	0.770	<0.01	0.739	<0.01	<0.01	<0.01

**Table 2:** Semi-quantitative analysis of B7-H4 expression in the examined tissues.

Percentage of Cells Showing Reactivity (%)	Staining Pattern	Stage
<1	No reactivity	0
1–20	Any	1
21–50	Low	2
>50	Low	3
>20	High	3

## Data Availability

Data available on request.
